# Barriers to Accessing Video-Based Telehealth Appointments at a Community Veterinary Clinic During the COVID-19 Pandemic

**DOI:** 10.3389/fvets.2022.878220

**Published:** 2022-07-19

**Authors:** Alena M. Naimark, Stella E. Elwood, Emily McCobb, Benjamin Kragen, Erin K. King, Greg Wolfus

**Affiliations:** ^1^Section of Community Medicine, Department of Clinical Sciences, Cummings School of Veterinary Medicine, Tufts University, North Grafton, MA, United States; ^2^Heller School for Social Policy and Management, Brandeis University, Waltham, MA, United States

**Keywords:** video-chat, digital access, community veterinary medicine, Tufts at Tech, digital divide, access to veterinary care

## Abstract

**Objective:**

To assess veterinary needs of clients with low socioeconomic status during the COVID-19 pandemic, to explore the impact of the pandemic on marginalized communities, and to understand perceptions regarding barriers and incentives of telehealth appointments as a method to increase care access.

**Sample:**

205 active Community Veterinary Medicine Clinic clients at Tufts at Tech Community Veterinary Clinic in Worcester, MA.

**Procedures:**

This cross-sectional study used a survey-based method to assess veterinary needs of clients with low socioeconomic status during the COVID-19 pandemic. The survey measured client perceptions regarding potential barriers and incentives of telehealth appointments. Participants were randomly sampled from a list of active clients and completed the survey either by email or over the phone. The survey was available in both English and Spanish. Clients who completed the questionnaire received credit for a free examination.

**Results:**

A total of 205 clients submitted survey responses. Factors affecting access to veterinary care were impacted by the COVID-19 pandemic. Access to reliable internet in the home was reported by 87% (*n* = 177) of participants. Digital access was correlated with education when controlling for race, income, age, and gender. Education was predictive of comfort with using video-chat (χ^2^ = 65, df = 24, *p* ≤ 0.01) and of whether or not clients reported need for assistance for using video-chat (χ^2^ = 52, df = 18, *p* ≤ 0.01). Patient education level was significantly predictive of wanting to use telehealth for at least one type of appointment (*p* ≤ 0.05).

**Conclusions and Clinical Relevance:**

The findings indicate that implementing telehealth services at this Community Medicine Clinic is feasible for much of the active clientele and offers a new avenue to provide veterinary care during times of social distancing restrictions and beyond.

## Introduction

Improving access to veterinary care for underserved communities has become a recent priority of the veterinary medical profession, which is now working to understand and create programs to address barriers to care access. Analyzing these barriers is of specific relevance during the COVID-19 pandemic, when states across the nation have been forced to implement quarantines and shut down public businesses, amplifying already existing obstacles to care access. Prior to the COVID-19 pandemic, commonly identified barriers to veterinary care access included cost, accessibility, cultural/language barriers, veterinarian-client communication/relationships, and lack of client education (1). Certain barriers to care access such as accessibility were especially amplified during the COVID-19 pandemic shutdown. An airborne infectious disease outbreak of this magnitude has not occurred since the Spanish Flu outbreak in 1918, and there is no precedent for understanding the ways in which pandemic restrictions have impacted veterinary care access (2). Many underserved communities have experienced obstacles to veterinary care access even prior to the pandemic, and it is essential to learn how the pandemic has impacted these barriers to effectively address them.

Telehealth, especially in the form of video-based appointments, has been implemented and studied in human medicine as a method for increasing medical care access to underserved populations (3). Video-based telehealth appointments specifically allow for the provision of remote medical care, a necessity designated by the early restrictions of the COVID-19 pandemic. During the peak of the COVID-19 shutdown, from January to March of 2020, human telehealth visits increased by 50% compared to the previous year (4). Similar statistics are not available regarding telehealth use in veterinary medicine during the COVID-19 pandemic, and the uses and benefits of telehealth in veterinary medicine are much less studied and well-defined. A hypothesized benefit of offering video-based veterinary telehealth appointments both during the COVID-19 pandemic restrictions and beyond is to increase access to remote veterinary care for underserved populations, which is especially desirable during a time when everyday barriers are magnified by the restrictions of the pandemic.

To address barriers to care for low-income individuals in the community, Tufts University's Cumming School of Veterinary Medicine opened a full-service low-cost teaching clinic, Tufts at Tech Community Veterinary Clinic (TAT) in 2012 (5). To qualify as a client to receive services at Tufts at Tech, clients must either be part of the Supplemental Nutrition Assistance Program (SNAP), Women Infants and Children (WIC) program, live at an approved Worcester Housing Authority address, or have a current Worcester Technical High School student living at their current address. During the first wave of the pandemic in Massachusetts in the winter and spring of 2020, TAT was closed and subsequently only able to offer pre-scheduled surgical procedures. This significant reduction in services offered at one of only two community veterinary clinics in the Worcester region left most clients without an affordable alternative option for veterinary services. The pandemic thus served as a catalyst for exploring the possibility of telehealth implementation at TAT as a means of increasing care access. A recent article in *the Veterinary Record* highlights that telehealth services in the United Kingdom have been thriving during the pandemic and have provided clients with access to veterinary care, and doctors and clinics access to business (6). It is possible that telehealth services could show similar benefit locally in a community veterinary medicine setting.

Current examples of applications of telehealth in veterinary medicine include providing pet owners texting services in the aftermath of a natural disaster to facilitate contact with expert veterinarians, offering after hour tele-triage services by phone, and using videoconferencing for recheck appointments after high-quality, high-volume spay and neuter clinics in geographically isolated regions (7). A 2018 randomized clinical control trial evaluated client satisfaction of videoconferencing recheck appointments for routine surgical procedures and found that clients were equally as satisfied with telehealth recheck appointments as they were with in person rechecks (8). Findings suggested that telehealth appointments may be efficacious when it comes to specific types of follow up visits, however previous work has not examined how technology-dependent visits may be perceived and utilized differently by communities of different demographics.

It is important to note that video-chat based telehealth appointments and other technology dependent interventions may be more accessible to some communities than others. The term “digital divide” is a widely used term that describes the disparities in access to technology based on social determinants such as socio-economic status, race, education, and income that exist in the United States and globally. Non-white households, households with seniors, and lower-income households are demographics found to have the least access to computers and internet within the home. In addition, income and education are negatively correlated with access to computers and internet (9). These findings are consistent among a variety of studies, including a 2013 study that explored computer access in an urban, low-income community in California (10), and a 2015 study that found that older adults who are economically or socioculturally disadvantaged have less access to reliable internet (11). Based on these findings and the notion of a growing digital divide, it is expected that income and education level will be negatively correlated with access to technology among TAT clients.

To examine the impact of the pandemic and whether telehealth could be a useful tool to increase care access to community medicine clients at TAT, a client survey was developed. According to the 2021 AAHA/AVMA Telehealth Guidelines for Small Animal Practice and the Commonwealth of Massachusetts Board of Registration of Veterinary Medicine, an established Veterinary Client Patient Relationship (VCPR) is required to practice veterinary medicine both in person and remotely via telehealth (12, 13). Due to these guidelines, only current clients with existing VCPRs were surveyed. The survey- and more broadly, the present study- explored specific ways the pandemic has affected active TAT client access to veterinary care. Additionally, client access to, and opinions regarding telehealth and related technologies were assessed with the goal of determining feasibility of telehealth implementation as an alternative method of care in the context of community veterinary medicine.

## Materials and Methods

A survey questionnaire was used to assess the veterinary needs of community medicine clients with low socioeconomic status during the COVID-19 pandemic. The survey questions were developed in conjunction with a health economist and telehealth expert. Participants were randomly selected from a list of current and active clients at TAT. Active clients were defined as existing clients who have attended appointments with TAT between June 1, 2019 and June 1, 2020. A list of all active clients at TAT was generated from the electronic medical records system (EMR)^1^, and an initial list of 200 active clients was generated using a random number assignment on Excel. The generated list of clients was color coded and divided between two student researchers to avoid overlap.

### Study Design

This cross-sectional study examined patient access to and use of digital tools used for telehealth. Participants with email addresses listed in the EMR were sent an email that contained a brief English and Spanish description of the study along with a link to both an English and Spanish version of an online survey. Clients without an email address listed or those that had an inactive email address (denoted by a notification saying that the address was invalid) were called from an anonymous phone number via a Google Voice account associated with the research email. The participants were first asked their preferred language, and if they had an email address where the survey could be sent. If a student researcher who did not speak Spanish contacted a participant who preferred to answer survey questions in Spanish, the other student researcher was notified and proceeded with said client. If the client did not have a working email address or preferred to complete the survey over the phone, the student researcher recorded the responses. A consent statement was provided at the beginning of the survey, and by clicking “next” or verbally indicating yes over the phone, participants signaled consent.

If the client was not reached on the phone the first time, the researcher left a scripted message in the case of a working phone number. Client contact was attempted three times before a client was marked “unable to be contacted,” and replaced in the sampling randomization. For clients with email addresses, a reminder email was sent 2 weeks following the initial email to those who had not yet completed the survey. This entire sampling procedure and methods were repeated between the dates of June 1, 2020 and August 15, 2020 until a sample size of 848 clients were contacted, and 205 surveys were completed, yielding a 24% response rate.

All clients who completed the questionnaire received a free examination credit on their TAT account as a thank you for participation. All responses remained unidentified and confidential. The project was granted an exemption from review by the Tufts Institutional Review Board (Protocol # Number: 00000708).

### Measures

Data regarding participant demographics, including age, gender, race, maximum level of education achieved, annual household income, and whether a primary language other than English was spoken at home, were collected. Factors affecting access to veterinary care both before and during the COVID-19 pandemic, including finances, transportation, client physical or mental health condition, scheduling, difficulty getting pets out of the house, COVID fears, and others were assessed. Participant digital access use was also studied. Participant access to a working computer, smartphone, reliable internet, and email address was assessed. These four questions were consolidated into a single categorical variable, which demonstrated acceptable internal consistency (Cronbach's Alpha of 0.71) and produced a single strong factor with an eigenvalue of 2.34. Digital use was also evaluated. Participant comfort using video-chat was assessed using a Likert scale that ranged from very comfortable to very uncomfortable. Concerns using video-chat, the appeal of video-chat appointments, help navigating video-chat appointments, and interest in certain types of video-chat appointments, and willingness to pay for such appointments were additionally assessed.

### Statistical Analysis

Client responses were exported from Qualtrics into Excel spreadsheets and stored in a password protected cloud-based account (TuftsBOX). Data was cleaned, organized, and exported again into Stata 16 for statistical analysis. Descriptive statistics were calculated. Bivariate statistics were performed using Chi-squared test and Analysis of Variance (ANOVA) to compare sets of categorical and categorical/continuous variables, respectively. Multivariate statistics were performed using ordinary least squares regression to understand the relationship between digital use and income controlling for race, ethnicity, age, gender, income, and education. A logistic regression model was used to understand the relationship between having a working a computer and income, controlling for age, gender, income, and education.

## Results

### Survey Response Rate

In total, 848 clients were randomly sampled from a list of 1801 active Tufts at Tech clients. Of this, 680 clients (80%) were contacted via email, and 168 (20%) did not provide an email and were contacted via phone. Out of the 848 clients sampled, 68 (8%) did not have an email address provided on the active client spreadsheet but provided it over the phone so a survey could be disseminated. A total of 205 participants responded to the survey, for an overall response rate of 24%. Out of the 205 participants, 164 (80%) responded after receiving an email and 30 (15%) responded verbally over the phone. The remaining 11 (5%) participants returned a survey without being contacted directly by a researcher.

Of the 680 clients sampled who listed an email address, 24% completed the survey, and of the 230 total clients who received phone calls, 13% completed the survey. Due to temporarily or permanently disabled phone numbers, 38 (4%) of the sampled clients were unable to be contacted by phone or email.

A total of 32 of the 848 contacted clients (4%) were contacted but were not qualified to participate in the study due to either having no active patient (patient deceased) or not having attending a scheduled appointment in 2019.

### Participant Demographics

Most participants were between the ages of 31–70 years old. A great majority (69%) of participants identified as White, English-speaking females. Over 50% of participants achieved a high school degree or less than a high a high school degree as their maximum level of education. Greater than half of the participants indicated a total annual household income of < $25,000. One hundred and twenty-five clients (8%) indicated owning a dog, 26 clients (16%) indicated owning a cat, and 6 clients (4%) indicated owning a small mammal or no pet at the time of taking the survey. Participant demographics can be found in Table 1.

**Table 1:** Demographics for 205 clients of a low-cost veterinary clinic in Worcester, MA.

**Variable number (*n*)**	**Percent**
**Age (187)**	
18–29	14%
30–49	44%
50–69	36%
70+	6%
**Gender (201)**	
Female	81%
Male	17%
Trans	0%
Non-binary	0.5%
Prefer not to answer	1.5%
**Maximum education achieved (202)**	
Less than high school	8%
High School	44%
Associates	23%
Bachelors	11%
Professional	8%
Prefer not to Answer	6%
**Race and ethnicity, can chose more than one (208)**	
Native American	0.5%
Black	3%
Pacific Islander	0%
White	69%
Latinx	21%
Asian	0.5%
Other	5%
Prefer not to answer	1%
**Language spoken other than english (193)**	
Yes	22%
No	78%
**Total household income (201)**	
< $25k	65%
$25k–$50k	16%
>$50k	3%
Prefer not to answer	16%

*$= US dollars*.

### Work During the Pandemic

When assessing client ability to work since the start of the pandemic, we found that 20 participants (10%) were able to work from home, 35 participants (17%) continued to work in person, 39 participants (19%) had been temporarily laid off, and 9 participants (4%) had been permanently laid off. A total of 97 participants (48%) indicated that they were not working before the pandemic, and 4 participants (2%) declined to answer the question.

### Client Reported Answers to Access to Care and Digital Access Questions

The most common factor clients reported to affect access to veterinary care before the COVID-19 pandemic was finances, while during the pandemic, the scheduling related to limited hours of operation was stated as the factor most impacting access to care (Table 2). The majority of participants indicated that their household had working computers, e-mail addresses and smartphones, as well as having access to reliable internet in the home. When asked about web application use for video-chatting in the past, most participants stated they had previously done so (Table 3).

**Table 2:** Survey responses from 205 clients of low-cost veterinary clinic in Worcester, MA regarding factors affecting access to veterinary care pre-pandemic and during the pandemic (can answer more than once).

**Variable**	**freq**.	**Percent**	**freq**.	**Percent**
	**Pre-pandemic (n)**		**During Pandemic (n)**	
Finances	115	42%	87	28%
Transportation	33	12%	20	6%
Client has a physical or mental health condition	29	10%	28	9%
Schedule	39	14%	123	40%
Difficult to get patient out of house	26	9%	15	5%
Other	2	1%	10	3%
Prefer not to answer	18	6%	12	4%
No problems	12	4%	11	4%
Covid fears	N/A		5	2%

**Table 3:** Digital Access Survey Responses from 205 Clients of a Low-Cost Veterinary Clinic in Worcester, MA.

**Variable (*n*)**	**Percent**
**Have a working computer (205)**	
Yes	71%
No	28.5%
Prefer not to answer	0.5%
**Have a smartphone (205)**	
Yes	88%
No	11.5%
Prefer not to answer	0.5%
**Have access to reliable internet in the home (203)**	
Yes	87%
No	11%
Prefer not to answer	1%
**Have an e-mail address (203)**	
Yes	93%
No	6%
Prefer not to answer	1%
**Have used a web application for video-chat (205)**	
Yes	81%
No	18%
Prefer not to answer	1%

### Client Reported Answers to Video-Chat Use Questions

Of the participants who had never used a video-chat platform in the past, reasons cited for not having done so include not knowing it was possible, not having a device that supports this function, not knowing how to set up the process, and not having had the need or want to do so. Less than half of participants stated great comfort in using video-chat, and 25% of participants indicated discomfort or never having used video-chat at all in the past. Almost 50% of participants indicated the definite need or potential need for assistance to navigate video-chat appointments. When asked about appealing types of appointments to attend over video-chat, clients indicated the most interest in attending video-chat appointments for medication refills or follow-up care. When participants were asked why video-chat appointments were appealing to them, the most common reasons stated were that it would save time traveling to and from the veterinary clinic, it would save time spent in the waiting room, and it would be easier for the patient. When compared to in-person appointments, over 50% of participants indicated a willingness to pay less for video-chat appointments (Table 4).

**Table 4:** Video-chat use survey responses from 205 clients of a low-cost veterinary clinic in Worcester, MA.

**Variable (*n*)**	**Percent**
**Comfort using video chat (203)**	
Very comfortable	40.5%
Somewhat comfortable	33.5%
Somewhat uncomfortable	6.5%
Uncomfortable	4.5%
Never used	15%
**Reasons for not video-chatting (162)**	
Did not know it was possible	2%
No video-chat capable device available	5%
Do not know how to set it up	9%
Have not had the need to/want to	5%
Other	1%
HAVE used video-chat before	77%
**Require assistance navigating video-chat**	
**(204)**	
Yes	23.5%
Maybe	26%
No	49.5%
Prefer not to answer	1%
**Why video-chat appointments are appealing**,	
**can choose more than one (529)**	
Not interested	9%
Save time traveling	21%
Save time waiting	18%
Save money on transportation	8%
Easier for pet	17%
Easier for me	18%
People I live with can participate in appointment	6%
Good during pandemic	1%
Other	2%
**Interest in video-chat appointment by type**,	
**can choose more than one (508)**	
None	8%
Skin	11%
Behavior	9%
Chronic condition	10%
Medication Refill	23%
Follow-up	20%
ER consultation	9%
Allergy	7%
Non-medication refill	1%
Other	2%
**Willingness to pay for video-chat**	
**appointment, relative to in-person**	
**appointment (150)**	
Less	51%
Same	34%
More	1%
Nothing	7%
Prefer not to answer	7%

### Bivariate and Multivariate Analysis

#### Factors Affecting Access to Veterinary Care Before and During COVID

Travel, finance, and timing related factors affecting access to veterinary care changed significantly during the COVID-19 pandemic (χ^2^ = 64, *df* = 7, *p* ≤ 0.001). Unsurprisingly, difficulty finding an appointment time during the pandemic contributed most to the Chi^2^ statistic (contribution to χ^2^ = 17). This indicates that a disproportionate number of patients reported difficulty getting an appointment as a barrier to access care. Interestingly, we also found that financial factors were not significantly associated with lack of access to care during the pandemic (Table 2).

#### Education as a Predictor of Digital Access and Video-Chat Use

No significant relationship was found between education level and having an e-mail address or owning a smartphone. However, education level was associated with owning a working computer (χ^2^ = 17, *df* = 5, *p* ≤ 0.004) and access to reliable internet (χ^2^ = 13, *df* = 5, *p* ≤ 0.018). We found that education level was significantly positively correlated with comfort with using video-chat (χ^2^ = 65, *df* = 24, *p* ≤ 0.01) and positively correlated with whether the clients reported the need for assistance for using video-chat (χ^2^ = 52, *df* = 18, *p* ≤ 0.001). We found that the combination variable of digital access was significantly positively correlated with education (*p* = 0.003) and significantly negatively correlated with age (*p* ≤ *0.00)* when controlling for sociodemographic variables including race, income, and gender, as displayed in the linear regression (Table 5).

**Table 5:** Linear Regression model showing the combination variable “Digital Access” as related to Sociodemographic Variables in a Population of 205 Clients of a Low-Cost Veterinary Clinic in Worcester, MA.

**Variable**	**Coef**.	***P* > *t***
Income	0.043	0.214
Race	−0.0418	0.054
Educate^*^	0.046	0.003^*^
Age^*^	−0.005	0.000^*^
Gender	0.038	0.305
_cons	0.958	0.001

*R^2^ = 0.182, adj. R^2^ = 0.155, and root MSE = 0.219*,

* *= sig finding*.

#### Income as a Predictor of Digital Access and Video-Chat Use

No significant relationship was found between client household income and having a smartphone, e-mail address, or access to reliable internet. Annual household income was associated with client reported ownership of a working computer (χ^2^ = 7.53, *df* = 2, *p* ≤ 0.023). This relationship became insignificant when we ran a logistic regression model that controlled for gender, race and ethnicity, education, and age. However, there was a significant difference in digital access across income levels (*f* = 3.92, *dfn* = 2, *dfd* = 166, *p* ≤ 0.01). This relationship similarly became insignificant when we controlled for sociodemographic variables.

#### Education Level as a Predictor of Disinterest in Telehealth

Twenty-one percent of participants indicated no interest in video-chat appointments, regardless of appointment type (Table 4). Education level was predictive of disinterest in telehealth appointments (*p* ≤ 0.05). Race, income and age were not significantly predictive of disinterest in telehealth appointments (*p* ≤ 0.079 and *p* ≤ 0.093 respectively).

## Discussion

Understanding client access to technology and comfort with digital use is an essential step in determining if video-based telehealth appointments can feasibly increase access to care in a community medicine clinic. When examining access to technology, most participants surveyed had access to a smartphone or computer. Furthermore, the fact that most participants indicated access to reliable internet in their homes suggests that the client population largely has access to the technology needed to support remote video-chat appointments.

As the data suggests, most clients had some experience and comfort using a video-chat platform, while just over half of clients reported the potential need for assistance navigating a video-chat appointment (Table 4). Exploring the specific types of assistance clients may need to effectively navigate a video-based telehealth appointment would be helpful to understand the resources and guidance the clinic could provide to streamline the appointment process. Of the clients who have not used video-chat before, reasons include never having needed to, not knowing how to set it up, or needing assistance setting it up (Table 4). If video-chat appointments were offered and accessible to clients, creating a “how-to-set-up-video-chat” training infographic or video clip for clients to review before navigating the process might prove useful. Furthermore, Health and Human Services has a website (teleahealth.hhs.gov) that provides information for both healthcare providers and clients about how to access and troubleshoot telehealth platforms, and would serve as a useful tool to guide telehealth users.

In addition to saving time traveling to and from the veterinary clinic, saving time in the waiting room, and being easier for the patient, clients also state that the limited hours and services offered at the clinic during the COVID-19 pandemic have greatly impacted ability to receive veterinary care (Table 4). Multiple recent studies that examined the impact of COVID-19 on veterinary care also corroborate this finding as a barrier to care access (14–16). Implementing remote video-chat appointments is a way to reduce this barrier. However, clients report concern regarding the quality of video-chat appointments when compared to in-person visits. If appointment types that are conducive to using video-chat, such as follow-up visits, chronic care management, medication refills, and others that do not require hands-on procedures are offered, it is possible that appointment quality will not be impacted and thus will become less of a concern, while still addressing and increasing care access. In fact, participants indicated interest in a variety of video-chat appointments for conditions that do not require hands-on procedures. It is important to note that almost one quarter of clients were not interested in any type of video-chat appointment (Table 4). This highlights the fact that telehealth is not an appealing option for everyone, even in cases where it may be logistically feasible.

An interesting question that was explored in this study was if there was a difference in factors affecting access to veterinary care before and during the pandemic. Before COVID-19, financial limitations were noted as the primary barrier to veterinary care access. Financial concerns were listed as a major barrier to care access by 64% of clients before the pandemic, while only 44% of clients cited this concern during the pandemic (Table 2). The chi2 test found that financial factors were less likely to be associated with access to care during the pandemic, while lack of services was more likely to be a barrier to care access during the pandemic. The financial difference may be a result of the $1,200 stimulus check that was distributed to Americans with a yearly income that was < $99,000 as part of the CARES act (17), or financial burden may have become less relevant with complete clinic closure and inability to access care. The fact that the clinic was initially only open for emergencies and special procedures and subsequently opened only for a finite number of appointments per day to comply with social distancing and staffing concerns left many clients without access to affordable veterinary care. Even if clients were *able to afford* veterinary care during the pandemic, it was an inability to access this care that became the major barrier. Offering telehealth appointments could increase the number of daily appointments offered, in a safe and practical manner.

The results indicated that a lower education level was predictive of disinterest in telehealth appointments, while race, income and age were not predictive of disinterest (Table 4). It is possible that other demographic variables may be predictive of disinterest in telehealth with a large sample size and should be further explored. Of those clients interested in video-chat appointments, telehealth appointments for medication refills and follow-up visits were among the most popular appointment types that clients were interested in attending remotely. Although not listed as an original option, some clients stated interest in telehealth appointments for non-medication refills, such as food or supplements (Table 4).

When examining patient demographics, it is essential to note that 65% of participants indicating making < $25,000 per year (Table 1), an expected finding given the qualification criteria for TAT. Anecdotal evidence from conducting surveys verbally over the phone suggest that many clients make significantly < $25,000 per year. Since most clients indicated making < $25,000 per year, a figure also supported by a study of TAT client demographics in 2018, including income brackets in increments lower than $25,000 may help to determine a more accurate understanding of client annual income and how income relates to digital access (18).

Additionally, most survey participants indicate an unwillingness to pay as much for a telehealth appointment when compared to an in-person visit (Table 4). This contrasts with a recent study conducted by Widmar et. al., that examined the willingness of pet owners to pay for veterinary telehealth appointments. Dog and cat owners from this study both indicated a willingness to pay more (approximately $38 for telehealth appointment with their own vet, and approximately $13 for telehealth appointment with another local vet) for video-chat appointments (19). In the Widmar et.al. study, only 24% of respondents had an annual income of < $24,999, compared to the 65% of participants in this income bracket in the present study. At TAT, in-person appointments cost $16. Offering telehealth appointments at a reduced cost when compared to in-person visits should be considered, however, if benefits of remote appointments such as no cost for transportation and no time spent in the waiting area were highlighted to the client, clients may be more willing to pay an equal amount for telehealth appointments as for in-person care. These phenomena should be explored with future research.

When considering the logistics of video-chat appointments, we must consider, and when possible, offer appointments in a client's first language. Over 20% of the clients surveyed indicated speaking a language other than English at home. Spanish accounted for the majority of this 22%, with Armenian and Russian also contributing significantly to this percentage. This demographic breakdown matches well with the 21% of the participant population who self-identified as Latinx (Table 1). Since almost one quarter of the clients speak Spanish as a primary language, offering telehealth appointments in both Spanish and English should be taken into serious consideration for program development.

This study had several limitations. Regarding formatting in the initially distributed emails, the English version of the text and the link to the English survey were placed before the Spanish versions. The researchers noted a lower-than-expected response rate to the Spanish survey after the first few weeks, and even found that Spanish speaking clients were completing the English version of the survey. Based on this observation, the researchers decided to re-format future emails so that English and Spanish links were located right next to each other. The initial placement of the Spanish link may have impacted how people responded to the English survey if it was not their native language. In fact, 22% of people who completed the survey *in English* indicated that they primarily spoke a language other than English at home, indicating that language barriers may have impacted survey responses.

Another important consideration is the terminology used in survey questions. When analyzing the maximum level of education that the participant population has achieved, over 50% of participants had either never completed high school or completed high school as their highest level of education. The questionnaire provided to clients was analyzed by a literacy checker, scoring a 68.9/100 on the Flesch Reading Ease Readability Formula, equivalent to a fifth-grade reading level. Although 86% of participants had a high school education or higher, this may not be indicative of literacy level or exposure to certain terminology. For example, the term “smartphone” was used in a question interested in understanding how many clients had access to this type of phone. Anecdotal evidence from surveys conducted over the phone suggest that some clients were not familiar with the term “smartphone,” and therefore indicated not having a smartphone. Yet, if a researcher changed the wording to “an iPhone” or “an Android,” clients would then change their answer to indicate in the affirmative. Additionally, clients were only asked if they had access to working computers. The survey did not evaluate access to other devices such as tablets that have the capacity to support a video-chat platform. It is therefore possible, and even likely, that >71% of clients have a working computer (in the form of a laptop or tablet of some type), and >88% of clients have a smartphone, indicating widespread access to technology across the sample population.

Voluntary response bias likely impacted the results of this study. As an incentive for participation, clients were offered an exam credit for one of their pets. This incentive likely influenced the population of clients who decided to participate in the study. Clients that needed veterinary care, and those that were struggling financially could have been more likely to respond due to the offered incentive. Although the monetary value of the incentive ($16) was relatively small, this was a significant incentive for our socio-economically disadvantaged participants. Clients who were not in need of an exam credit may have been less inclined to respond, therefore skewing the participant population toward those with worse financial instability and/or a need for veterinary care. Furthermore, 11 participants (5% of the sample population) returned a survey without ever being contacted by a researcher. Since surveys were de-identified upon completion to ensure client confidentiality, the surveys from these individuals were unable to be identified, and were therefore included in data analysis and impacted the findings of this study.

Clients who were unable to be reached by phone or by email did not participate in the survey. Excluding this population from the present study ignores a population of people who also face great barriers to veterinary care access. This subset of the population may not have access to smartphones, computers, or email addresses, making the option of telehealth appointments harder to access for a population already facing obstacles to care access. Additionally, it is essential to note that the sample population was sourced from a list of clients who were able to receive care at the clinic in the last year, so by nature those surveyed have been able to access veterinary care at least once in the past. Those who have greater barriers to care access and were not seen in the past year, potentially due to such barriers, were not included in the sample population, potentially skewing the results. This sample bias is a significant limitation to the study. It is essential to consider this challenging-to-access population of people when implementing alternative forms of veterinary medicine appointments, and to find reasonable options that suit the variety of challenges our clients face.

The findings of this study indicate that implementing telehealth services as a means of increasing access to veterinary care is possible when considering client access to technology. Most clients have access to a smartphone or computer and express at least some level of comfort with interacting with video-chat platforms. Maximum level of education was found to correlate with both owning a working computer and access to reliable internet and should be taken into consideration when implementing telehealth services and navigating the digital divide. Although telehealth appointments may help increase access to veterinary care for many clients, other interventions must be considered to reach populations for whom access to video-chat technology is difficult or logistically impossible.

## Data Availability Statement

The raw data supporting the conclusions of this article will be made available by the authors, without undue reservation.

## Ethics Statement

The studies involving human participants were reviewed and approved by Tufts University Social and Behavioral Sciences Human Subject Institutional Review Board. Written informed consent for participation was not required for this study in accordance with the national legislation and the institutional requirements.

## Author Contributions

AN, SE, and EM conceived and designed the project with advice from EK and GW. Data were collected and analyzed by AN, SE, and BK. The final paper was written by AN and edited by all authors. All authors contributed to the article and approved the submitted version.

## Funding

This project was supported and made possible by grants from PetSmart Charities (Grant # PR022) and the Tisch Student Covid Response Program.

## Conflict of Interest

The authors declare that the research was conducted in the absence of any commercial or financial relationships that could be construed as a potential conflict of interest.

## Publisher's Note

All claims expressed in this article are solely those of the authors and do not necessarily represent those of their affiliated organizations, or those of the publisher, the editors and the reviewers. Any product that may be evaluated in this article, or claim that may be made by its manufacturer, is not guaranteed or endorsed by the publisher.

## Footnotes

^1^StringSoft Veterinary Practice Management Software Version 20.2.4000.10000.
